# Single-molecule imaging of a three-component ordered actin disassembly mechanism

**DOI:** 10.1038/ncomms8202

**Published:** 2015-05-21

**Authors:** Silvia Jansen, Agnieszka Collins, Samantha M. Chin, Casey A. Ydenberg, Jeff Gelles, Bruce L. Goode

**Affiliations:** 1 Department of Biology, Rosenstiel Basic Medical Science Research Center, Brandeis University, 415 South street, Waltham, Massachusetts 02454, USA; 2 Department of Biochemistry, Brandeis University, 415 South street, Waltham, Massachusetts 02454, USA

## Abstract

The mechanisms by which cells destabilize and rapidly disassemble filamentous actin networks have remained elusive; however, Coronin, Cofilin and AIP1 have been implicated in this process. Here using multi-wavelength single-molecule fluorescence imaging, we show that mammalian Cor1B, Cof1 and AIP1 work in concert through a temporally ordered pathway to induce highly efficient severing and disassembly of actin filaments. Cor1B binds to filaments first, and dramatically accelerates the subsequent binding of Cof1, leading to heavily decorated, stabilized filaments. Cof1 in turn recruits AIP1, which rapidly triggers severing and remains bound to the newly generated barbed ends. New growth at barbed ends generated by severing was blocked specifically in the presence of all three proteins. This activity enabled us to reconstitute and directly visualize single actin filaments being rapidly polymerized by formins at their barbed ends while simultanteously being stochastically severed and capped along their lengths, and disassembled from their pointed ends.

## Abstract (web summary)

The roles of Coronin, Cofilin and AIP1 in promoting actin disassembly have not been well understood. Here using single-molecule fluorescence imaging, Jansen *et al*. show that the three proteins act together in a coordinated, temporal pathway to induce rapid severing and disassembly of actin filaments.

## Graphical abstract

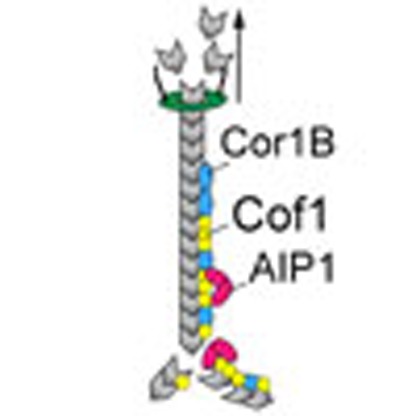

Actin is a highly abundant cytosolic protein and polymerizes to form dynamic filamentous networks that drive many biological processes, including cell morphogenesis, cell motility, endocytosis, and intracellular transport. Dynamic rearrangements of the actin cytoskeleton are achieved through controlled nucleation and elongation of filaments from a finite pool of ATP-actin monomers. This is counterbalanced by rapid destabilization and disassembly of aged (ADP) filaments, which serves to both replenish the actin monomer pool and sculpt network geometry. We now have a mature understanding of how actin filament arrays are initially formed in cells, revolutionized by the elucidation of the molecular mechanisms underlying actin filament nucleation and elongation^1^,^2^. In contrast, our understanding of how these networks are disassembled remains highly incomplete. The only well understood step in the disassembly process is filament severing. In a wide range of organisms this step is mediated by ADF/Cofilin (hereafter referred to as Cofilin), which binds cooperatively to the sides of ADP-actin filaments and induces structural changes, leading to breaks between the Cofilin-decorated and undecorated regions^3^,^4^,^5^,^6^,^7^. Severing amplifies the number of filament ends from which subunit dissociation can occur, thus reducing the time to full depolymerization.

Despite these advances in our knowledge of how Cofilin severs filaments, a number of critical questions have remained unanswered. First, Cofilin alone binds surprisingly slowly to ADP-actin filaments (*k*_on_=0.013 μM^−1^ s^−1^), suggesting that additional cellular factors may be required to enhance Cofilin recruitment to F-actin for inducing rapid actin disassembly^8^,^9^. Second, even though Cofilin is present at concentrations of 5–20 μM in mammalian cells^10^,^11^,^12^, maximal severing by Cofilin *in vitro* was observed at nanomolar concentrations, and micromolar concentrations of Cofilin perplexingly led to overdecoration and stabilization of filaments^8^,^13^. Third, it has been unclear how actin polymerization is prevented at the new barbed ends of filaments generated by severing. Cells maintain high levels of ATP-actin monomers, ∼2 orders of magnitude above the critical concentration for barbed end assembly, and thus cytosolic conditions strongly favour actin assembly over disassembly^14^,^15^. As a consequence, severing without simultaneous capping of barbed ends will result in net growth rather than disassembly^8^,^10^. Together, these observations suggest that additional cellular factors must work in concert with Cofilin to achieve highly efficient severing and disassembly *in vivo*.

Mounting genetic and biochemical evidence has implicated three proteins (Srv2/CAP, AIP1 and Coronin) in functioning with Cofilin to promote actin disassembly *in vivo*. Yeast and mammalian homologues of Srv2/CAP facilitate Cofilin-mediated actin disassembly by enhancing filament severing 4–8 fold^16^,^17^,^18^,^19^,^20^. However, the roles of AIP1 and Coronin are less well understood. AIP1 binds to F-actin and Cofilin, but it has remained controversial whether AIP1 enhances Cofilin-mediated severing and/or caps the barbed ends of filaments after severing^21^,^22^,^23^,^24^,^25^. The role of Coronin has been even more enigmatic, with ostensibly conflicting genetic and biochemical observations. While genetic data strongly support a role for Coronin in promoting actin turnover, purified yeast and mammalian Coronins both inhibit rather than enhance Cofilin-mediated severing *in vitro*^26^,^27^,^28^,^29^. Thus, the functions and mechanisms of AIP1 and Coronin in regulating actin disassembly have remained poorly understood.

One of the important advances in our understanding of actin disassembly mechanisms came from the recent biochemical work of Brieher and co-workers^30^,^31^, who isolated Cofilin, AIP1 and Coronin from cell extracts as factors that together induce the disassembly of *Listeria* actin tails or purified actin filaments even under assembly-promoting conditions. These studies linked together for the first time the functions of AIP1 and Coronin in Cofilin-mediated actin disassembly. Here we have employed multi-wavelength total internal reflection fluorescence (TIRF) microscopy to directly observe Cofilin, AIP1 and Coronin during actin filament severing and disassembly, and thus define better the roles of each protein and their collective mechanism for inducing rapid actin disassembly. Our study reveals that these three proteins work in a temporally ordered manner to rapidly disassemble F-actin. This process is initiated by binding of Coronin to filaments, which in turn greatly enhances Cofilin recruitment to filaments sides. Last to arrive is AIP1, which invariably triggers severing. After inducing severing, AIP1 remains bound to the newly generated barbed ends, and together with Cofilin and Coronin blocks new growth, thus enabling filament disassembly even under assembly-promoting conditions.

## Results

### Rapid actin filament severing by a three-component mixture

**Figure 1:**
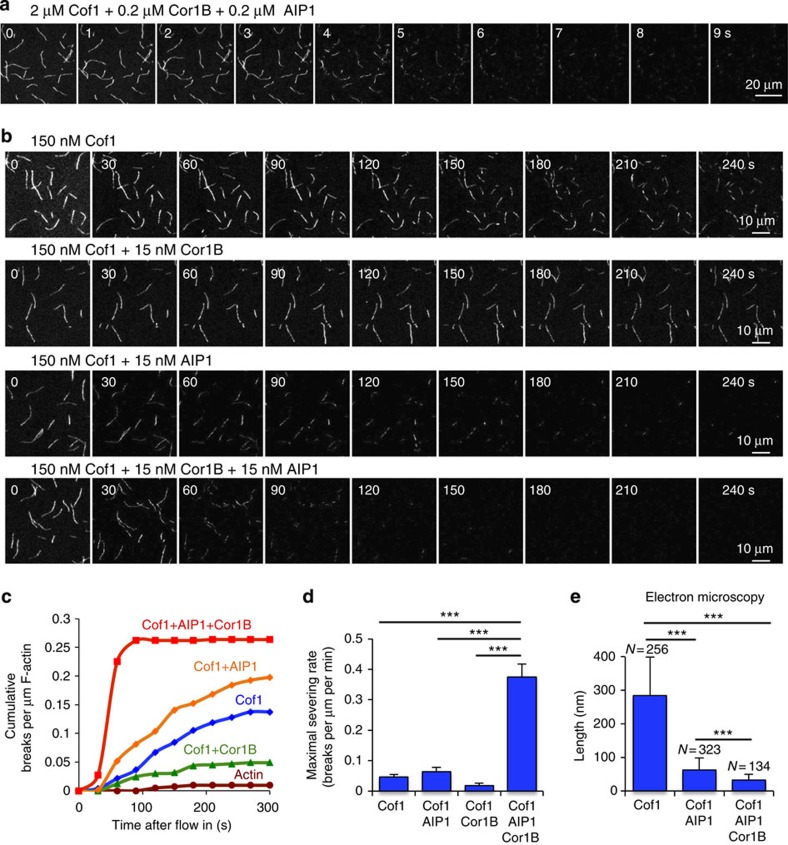
High rates of actin filament severing in the combined presence of mammalian Cof1, Cor1B and AIP1. (**a**) Time points from TIRF microscopy (Supplementary Movie 1) of pre-polymerized OG-labelled actin filaments after flowing in a mixture of the indicated concentrations of Cof1, Cor1B and AIP1. (**b**) Time points from TIRF microscopy (Supplementary Movie 2) as above, except flowing in lower concentrations (as indicated) of Cof1, Cor1B and/or AIP1. (**c**) Analysis of filament severing kinetics from TIRF assays performed as above. Each data point is the cumulative number of severing events per micron of filament at that time point, averaged for at least 60 filaments pooled from 3 independent trials of at least 20 filaments each. (**d**) Maximal rates of severing for each condition were determined by averaging the slopes of curves from three independent trials in the time interval from 30 to 60 s. Slope measurements at later time points confirmed that these were the maximal rates during the 300-s observation window. Error bars, s.d. (**e**) Electron microscopy of actin filament severing products. F-actin (2 μM) was incubated with different combinations of 2 μM Cof1, 0.2 μM Cor1B and 0.2 μM AIP1, then imaged by negative stain electron microscopy (examples of images in Supplementary Fig. 2). Average filament length (±s.d.) is graphed with the number of filaments analysed (*N*) above each bar. Statistical significance and *P* values for **d**,**e** were determined by analysis of variance and Tukey’s multiple comparison test; ***P<0.05.

To investigate how mammalian Cofilin-1 (referred to herein as Cof1), AIP1 and Coronin-1B (referred to herein as Cor1B) work together to regulate actin filament disassembly, we first compared their individual and combined effects on severing of surface-tethered Oregon-green (OG) labelled filaments in TIRF assays. Using low micromolar concentrations of Cof1 combined with 10-fold lower concentrations of Cor1B and AIP1 (approximating the ratio found in cells^29^,^30^,^32^), we observed complete severing and disassembly of filaments (10–15 μm in length) only 5 s after flow-in (Fig. 1a; Supplementary Movie 1). This precluded quantitative measurement of severing rates; therefore we reduced the concentrations of Cof1, AIP1 and Cor1B while maintaining the 10:1:1 ratio (150 nM, 15 nM and 15 nM, respectively), enabling differences in severing rates to be quantified. Under these conditions (Fig. 1b,c; Supplementary Movie 2; also see Supplementary Fig. 1), Cof1 alone induced an average of two to three severing events per filament during the 300 s observation window. Further addition of AIP1 led to a modest but significant (*P*<0.05, one-way ANOVA) increase in severing rate over the 300-s observation period (1.9±0.2 × 10^−4^ breaks per μm per s for Cof1 versus 3.4±0.45 × 10^−4^ breaks per μm per s for Cof1+AIP1, *n*=3). In contrast, Cor1B strongly and significantly (*P*<0.05, one-way ANOVA) inhibited severing by Cof1 (0.5±0.1 × 10^−4^ breaks per μm per s, *n*=3), consistent with its previously reported inhibitory effects in bulk assays^26^,^28^. In the absence of Cof1, AIP1 and Cor1B each showed no severing activity (Supplementary Fig. 1). The most striking effects though were observed in the combined presence of all three proteins (Cof1, AIP1 and Cor1B), where greatly enhanced severing was observed at all time points (Fig. 1c), and the maximal severing rate (measured between 30 and 60 s after flow-in) was 10-fold higher for the 3-component mixture compared with Cof1 alone (Fig. 1d).

TIRF microscopy only provides limited information about the size of F-actin severing products, since many of them are below the resolution of light microscopy, and some of the untethered fragments diffuse away. Therefore, we incubated filaments for 10 min with Cof1 alone or the combination of Cof1, AIP1 and Cor1B and examined the severing products by electron microscopy (Fig. 1e; also see Supplementary Fig. 2). Filaments incubated with Cof1 alone had an average length of 284±115 nm (*n*=256), compared with control filaments of 6,184±533 nm (*n*=19). Strikingly, filaments incubated with Cof1 and AIP1 had an average length of only 54±18 nm or ≈20 actin subunits (*n*=323). Filaments incubated with Cof1, AIP1 and Cor1B were even shorter, with an average length of 32±18 nm or ≈12 actin subunits (*n*=134). Taken together with our TIRF analysis, these data show that while AIP1 alone only modestly increases the initial rate of Cof1-mediated severing (Fig. 1c,d), it dramatically reduces the average size of the severing end products after prolonged incubation. In contrast, the contribution of Cor1B appears to be primarily to increase the initial rate of severing by Cof1 and AIP1 (Fig. 1c,d), while it only modestly affects the average size of the severing end products (Fig. 1e).

### Coronin accelerates Cofilin binding to actin filaments

Next, we fluorescently labelled Cof1, AIP1 and Cor1B, so that we could observe each of them in real time during actin filament disassembly by multi-wavelength TIRF microscopy. For Cof1, we reengineered its surface residues to leave only one exposed cysteine at a position where Cy3-maleimide labelling did not interfere with function (Supplementary Fig. 3). For AIP1 and Cor1B, SNAP tags were introduced at their C termini, and the resulting fusion proteins were directly labelled with benzyl guanine-conjugated dyes in the far-red spectrum (see Methods and Supplementary Fig. 3). The resulting proteins, Cy3–Cof1, Cor1B–SNAP649 and AIP1–SNAP647, had actin disassembly activities similar to unlabelled counterparts (Supplementary Fig. 3b–d).

**Figure 2:**
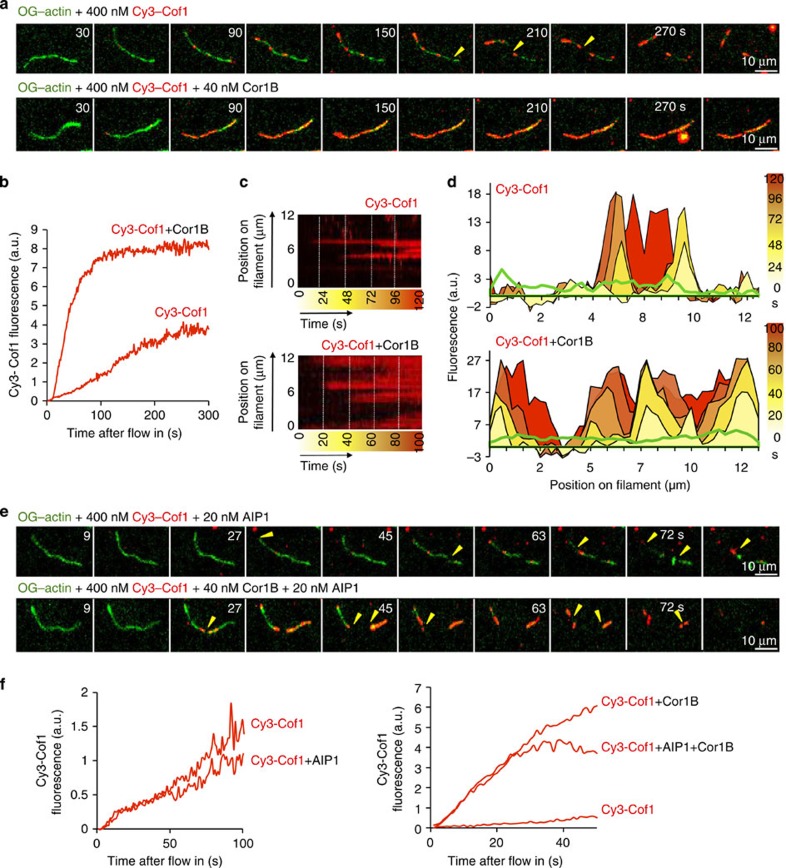
Cor1B dramatically increases binding of Cy3–Cof1 to actin filaments. (**a**) Time points from TIRF movies comparing binding of Cy3–Cof1, in the presence and absence of Cor1B, to preassembled OG-labelled actin filaments. Severing events are indicated by yellow arrowheads. (**b**) Kinetics of total Cy3–Cof1 fluorescence accumulation on actin filaments in the presence and the absence of Cor1B. The traces are averages from 3 independent trials (analysing 10–15 filaments each). (**c**) Kymographs each showing Cy3–Cof1 decoration of a single actin filament in the presence or absence of Cor1B. (**d**) Spatiotemporal profiles of Cy3–Cof1 distribution along the same filaments as in **c**, with time points in seconds as colour coded in the heat bars at the far right. (**e**) Effect of AIP1 on Cy3–Cof1 binding to actin filaments in the presence of Cor1B. Due to enhanced severing in the presence of AIP1, Cy3–Cof1 binding could only be monitored for 60–90 s after flow-in. Severing events are indicated by yellow arrowheads. (**f**) Kinetics of Cy3–Cof1 fluorescence accumulation on actin filaments in the presence of AIP1, with or without Cor1B. Traces are averages from 3 independent trials (analysing 10–15 filaments each).

We first examined the kinetics of Cy3–Cof1 binding to OG–actin filaments. Cy3–Cof1 associated slowly with filaments, accumulating in patches that gradually increased in brightness, consistent with a cooperative binding mechanism (Fig. 2a,b; Supplementary Movie 3). Further, Cy3–Cof1 decoration led to filament severing, with breaks occurring between the decorated and undecorated regions (Fig. 2a, yellow arrows). These results using mammalian Cof1 are consistent with those reported for yeast Cof1 (ref. 7), suggesting that the fundamental mechanism by which Cofilin interacts with and severs filaments is conserved between yeast and mammals.

Addition of unlabelled Cor1B in TIRF reactions led to a dramatic increase in the rate of Cy3–Cof1 binding to actin filaments, and produced heavily decorated, hyper-stabilized filaments (Fig. 2a,b; Supplementary Movie 3). Kymographs revealed an increase in the Cy3–Cof1 spot density on filaments in the presence of Cor1B relative to that observed in the absence of Cor1B (Fig. 2c,d). Each Cy3–Cof1 patch followed a similar pattern, starting as a small dot and gradually increasing in intensity and often merging with other patches. These results show that Cor1B markedly accelerates Cy3–Cof1 binding to filaments. By comparison, AIP1 had little if any effect on the kinetics of Cy3–Cof1 binding to filaments (Fig. 2f; Supplementary Movie 3). However, AIP1 had a profound effect on severing in the combined presence of Cor1B and Cy3–Cof1, inducing rapid fragmentation of filaments, including those heavily decorated by Cy3–Cof1 (Fig. 2e,f). Quantification of Cy3–Cof1 recruitment to filaments in the presence of AIP1 and Cor1B showed that AIP1 does not alter the kinetics of Cy3–Cof1 recruitment by Cor1B (Fig. 2f; Supplementary Movie 3).

Taken together, these observations suggest the following: (1) Cof1 binding to actin filaments is one of the rate-limiting steps in severing; (2) Cof1 binding is accelerated greatly by Cor1B and (3) the presence of AIP1 strongly enhances severing after Cof1 decoration.

### Coronin binds rapidly to actin filaments preceding Cofilin

**Figure 3:**
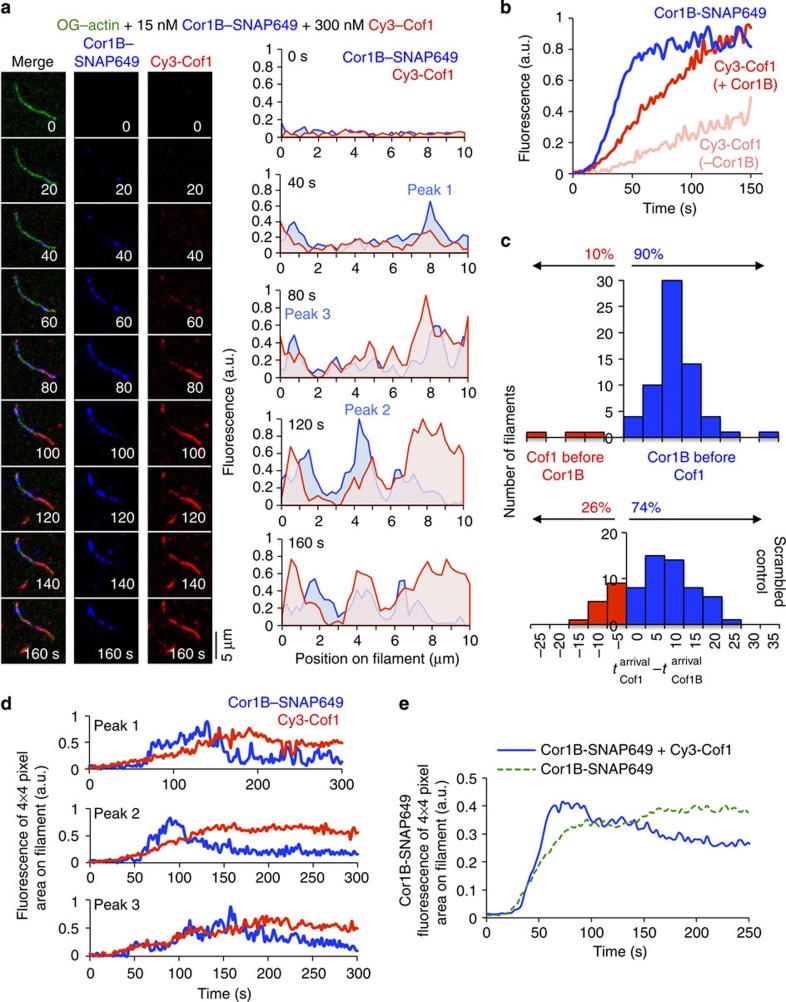
Simultaneous imaging of Cor1B–SNAP649 and Cy3–Cof1 binding to actin filaments. (**a**) Time points from a triple-colour TIRF movie (Supplementary Movie 4) showing binding of Cor1B–SNAP649 and Cy3–Cof1 to preassembled OG-labelled actin filaments after flow-in (time 0). Graphs show spatiotemporal profiles of Cy3–Cof1 and Cor1B–SNAP649 distributions along the same filament at different time points. (**b**) Kinetics of Cor1B–SNAP649 and Cy3–Cof1 fluorescence accumulation on the same set of actin filaments (blue and red traces). Traces are averages from 3 independent trials (analysing 10–15 filaments each). For comparison, the same analysis was performed for Cy3–Cof1 in the absence of Cor1B (faded red trace). (**c**) Distribution of time intervals between first appearance of Cor1B–SNAP649 and first appearance of Cy3–Cof1 on actin filaments (top panel). Data are from 3 independent trials (>20 filaments each). A control analysis in which randomly generated values were substituted in place of the observed times of Cor1B–SNAP649 binding yielded a similar distribution (bottom panel), indicating that Cy3–Cof1 had a high tendency to bind where Cor1B–SNAP649 was already bound. (**d**) Intensity profiles of Cor1B–SNAP649 and Cy3–Cof1 fluorescence of peaks (area of 4 × 4 pixels) indicated in the profiles in **a**. More examples are shown in Supplementary Fig. 4b,c. (**e**) Average Cor1B–SNAP649 fluorescence (derived from profiles as shown in **d** for three different regions of 4 × 4 pixels per filament, for 30 filaments in total (from three independent experiments) over the course of 300 s in the presence (blue curve) and absence (green curve) of Cof1.

To better understand the spatiotemporal relationship between Cor1B and Cof1 binding to filaments, we next performed three-colour TIRF microscopy to simultaneously observe binding of Cy3–Cof1 and Cor1B–SNAP649 to OG–actin filaments (Fig. 3a; Supplementary Movie 4). Cor1B–SNAP649 appeared on filaments rapidly, even faster than the appearance of Cy3–Cof1 accelerated by the presence of Cor1B–SNAP649 (Fig. 3b). Further, at the first observed binding location on a filament, Cor1B–SNAP649 almost always arrived ahead of Cy3–Cof1 (90% of the time; blue bars in upper panel Fig. 3c). As shown by a randomized control analysis (lower panel, Fig. 3c), this behaviour was not merely a coincidence, caused by faster appearance of Cor1B–SNAP649 on filaments. Instead, the data show that there was a tendency for Cy3–Cof1 to bind where Cor1B–SNAP649 was already bound. Further, we often observed that following Cy3–Cof1 recruitment, the Cor1B–SNAP649 signal at these sites would decline, suggesting that Cof1 might be able to displace Cor1B. Analysis of randomly chosen individual Cor1B–SNAP649 spots on filaments, showed that in the absence of Cy3–Cof1, the Cor1B–SNAP649 average fluorescence peaked and remained constant (Fig. 3e; Supplementary Fig. 4a,c). However, in the presence of Cy3–Cof1, the Cor1B–SNAP649 average fluorescence peaked and then slowly decreased, suggesting that accumulation of Cof1 may displace Cor1B–SNAP649 from these sites (Fig. 3d,e; Supplementary Fig. 4b). Altogether, these results suggest a preferentially ordered pathway, in which Cor1B typically binds to filaments first and then recruits Cof1 to the same sites, providing a means by which the otherwise slow association of Cof1 with filaments is greatly accelerated.

### AIP1 is recruited by Cofilin and triggers filament severing

**Figure 4:**
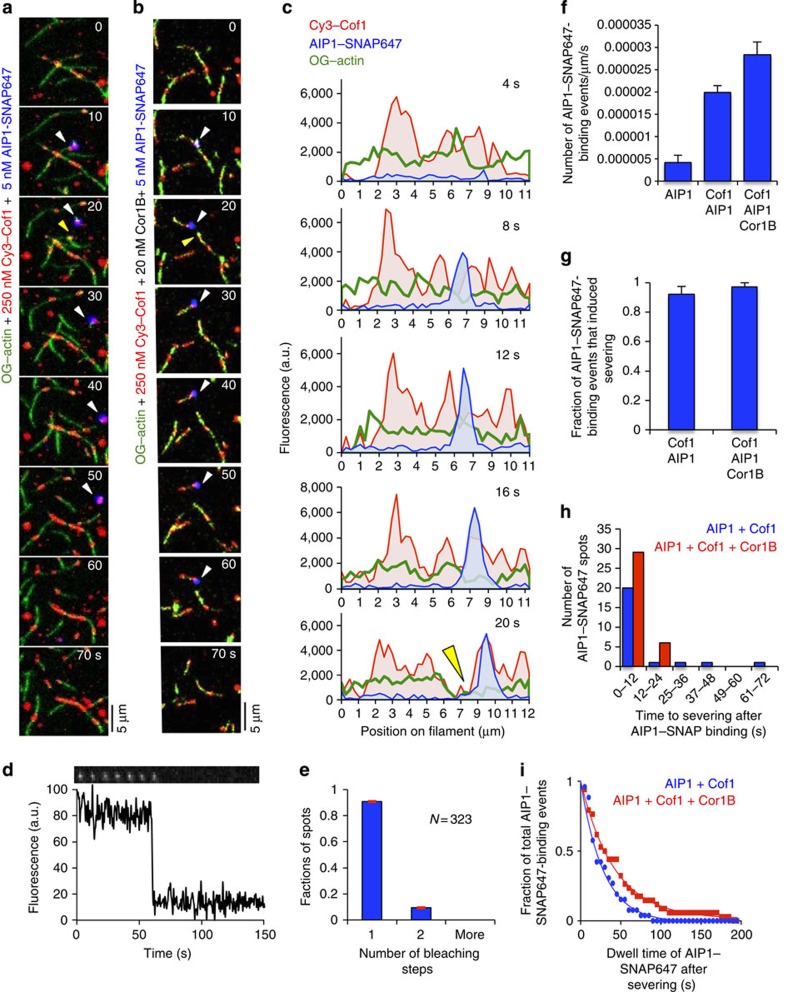
Recruitment of AIP1–SNAP647 to actin filaments induces rapid severing. (**a**,**b**) Time points from TIRF movies (Supplementary Movie 5) showing Cy3–Cof1 and AIP1–SNAP647 binding to preassembled OG-labelled actin filaments after flow-in, both in the absence **a** and presence **b** of Cor1B. White arrowheads indicate appearance of AIP1–SNAP647 fluorescence; yellow arrowheads indicate severing. (**c**) Cy3–Cof1 and AIP1–SNAP647 spatiotemporal profiles; same filament as in **b**. Green curve represents OG–actin fluorescence; yellow arrowhead indicates severing. (**d**) Stepwise photobleaching of surface-adsorbed AIP1–SNAP647. (**e**) Distribution of number of steps to complete photobleaching of AIP1–SNAP647 spots (±s.e., shown in red, *n*=323). (**f**) Frequency of binding of AIP1–SNAP647 (±s.e.m., *n*=3) on actin filaments for the indicated conditions. (**g**) Fraction (±s.e.m., *n*=3) of AIP1–SNAP647 binding events that induce actin filament severing. No statistically significant difference was observed by *χ*^2^-test (*P*>0.05). (**h**) Distribution of time intervals between appearance of AIP1–SNAP647 on filaments and severing. (**i**) Fitted curve for the distribution of AIP1–SNAP647 dwell times on actin filaments for the indicated conditions.

To investigate the spatiotemporal relationship of AIP1 and Cof1 binding to filaments, we performed three-colour TIRF microscopy experiments as above, except using AIP1–SNAP647, Cy3–Cof1 and OG–actin filaments, with and without unlabelled Cor1B (Fig. 4a–c, Supplementary Fig. 5 and Supplementary Movie 5). For these experiments, we used a low concentration of AIP1–SNAP647 (5 nM) to reduce background fluorescence in the TIRF images, which was required to enable single-molecule observations. We quantified the frequency of AIP1–SNAP647 binding events on filaments with and without Cof1. The AIP1–SNAP647 spots we observed on filaments were likely single molecules, as 90% of surface-adsorbed AIP1–SNAP647 spots photobleached in a single step (Fig. 4d,e). AIP1–SNAP647 binding to filaments was approximately fourfold higher in the presence of Cof1 and sixfold higher in the combined presence of Cof1 and Cor1B (Fig. 4f). These results are consistent with previous studies showing that AIP1 alone has low affinity for actin filaments (2–3 μM), but that its association with F-actin is enhanced by Cof1 (refs 22, 33, 34). We also observed that AIP1–SNAP647 binding always occured at sites where Cy3–Cof1 was already bound. Together, these observations suggest that Cof1 plays an important role in recruiting AIP1 to filament sides.

In addition, we observed that >90% of the observed events in which AIP1–SNAP647 appeared on filaments were rapidly followed by severing in the presence of Cof1, with or without Cor1B (Fig. 4g,h). The average time interval between binding of AIP1–SNAP647 and filament severing was 9 s (95% confidence interval (CI) (5, 17)) in the absence of Cor1B, and this did not change significantly in the presence of Cor1B (6 s (5, 8)). In most instances (56 of 63 events) AIP1–SNAP647 was also observed to remain bound to one of the new filament ends produced by severing (Fig. 4a–c, Supplementary Fig. 5 and Supplementary Movie 5). The average dwell time of AIP1–SNAP647 on severed ends was slightly longer in the presence of Cof1 and Cor1B (29.11 s; 95% CI (27.71, 30.66)) compared with Cof1 alone (19.54 s; 95% CI (18.72, 20.45)) (Fig. 4i).

### Capping at severed ends requires the three-component mixture

**Figure 5:**
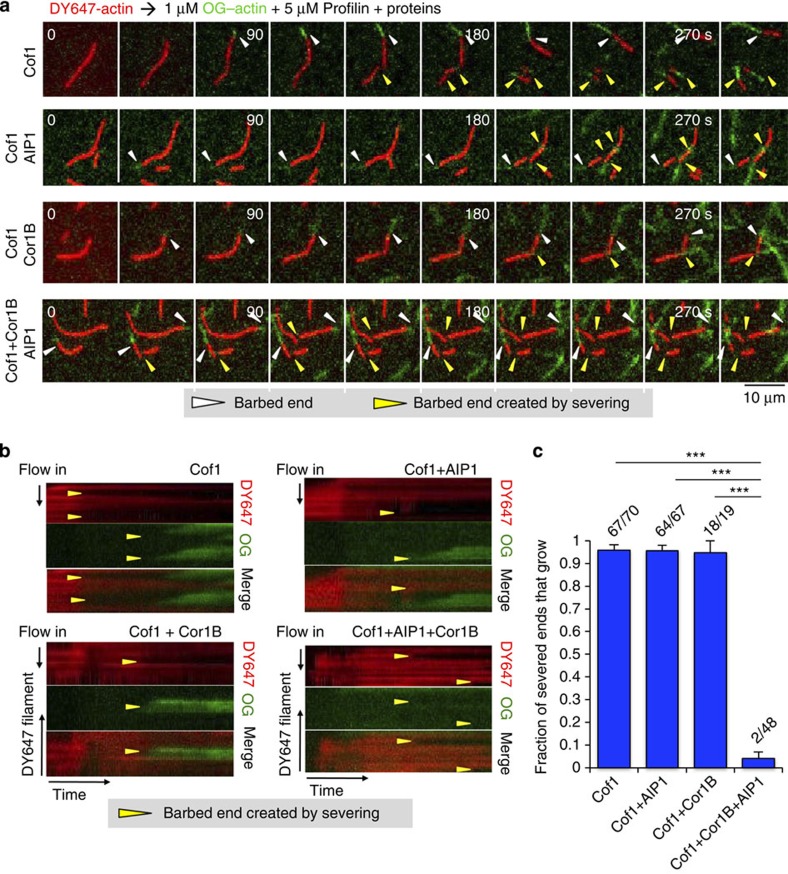
Growth at barbed ends generated by severing is blocked specifically by the combination of Cor1B, Cof1 and AIP1. (**a**) Time points from TIRF movies showing the polymerization of OG–actin (green) from the existing and newly generated barbed ends of preassembled, tethered DY647–actin filaments (red) after severing. After DY647–actin filaments were preassembled, the indicated proteins were flowed in, and both severing and new barbed end polymerization were monitored for 300 s. White arrowheads designate the original barbed ends; yellow arrowheads indicate new barbed ends generated by severing. (**b**) Kymographs showing DY647– and OG–actin fluorescence for the same filaments, severed by the indicated combination of proteins. (**c**) Fraction (±s.e.m., *n*=3) of newly generated barbed ends that grow within 300 s after flow-in. The fraction of filaments that grew after severing is shown above each bar. Statistical significance and *P* value were determined by a *χ*^2^-test; ***P<0.05.

As mentioned earlier (see Introduction), cytosolic conditions strongly favour actin assembly over disassembly because of the high concentration of ATP-actin monomers. For this reason, cells require a mechanism for efficient capping or blocking of new barbed ends generated by severing; otherwise, severing will promote increased growth rather than disassembly. To address whether Cof1, Cor1B and/or AIP1 can block actin polymerization at newly generated barbed ends after severing, we developed a two-colour actin assay, in which we first polymerized and tethered DY647-labelled actin filaments, then replaced the solution by flow-in with a mixture of OG–actin monomers, profilin and different concentrations of Cof1, Cor1B and/or AIP1 (Fig. 5; Supplementary Movie 6). Under each condition, the original barbed end (white arrows, Fig. 5a) continued to polymerize, indicated by the appearance of new (green) polymer. In the presence of Cof1, Cof1 and AIP1, or Cof1 and Cor1B, polymerization was also observed at nearly all of the barbed ends generated by severing (yellow arrows in Fig. 5a,b; Supplementary Movie 6). In striking contrast, polymerization was rarely observed at barbed ends generated by severing in the presence of Cof1, Cor1B and AIP1 (Fig. 5a–c; Supplementary Movie 6). These observations show that efficient obstruction of new growth at barbed ends generated by severing requires the combination of all three proteins—Cof1, Cor1B and AIP1. Further, they show that these proteins do not block polymerization at the original, growing barbed end, and thus their ability to inhibit barbed end growth may be closely coupled to severing.

### Reconstitution of rapidly treadmilling actin filaments

**Figure 6:**
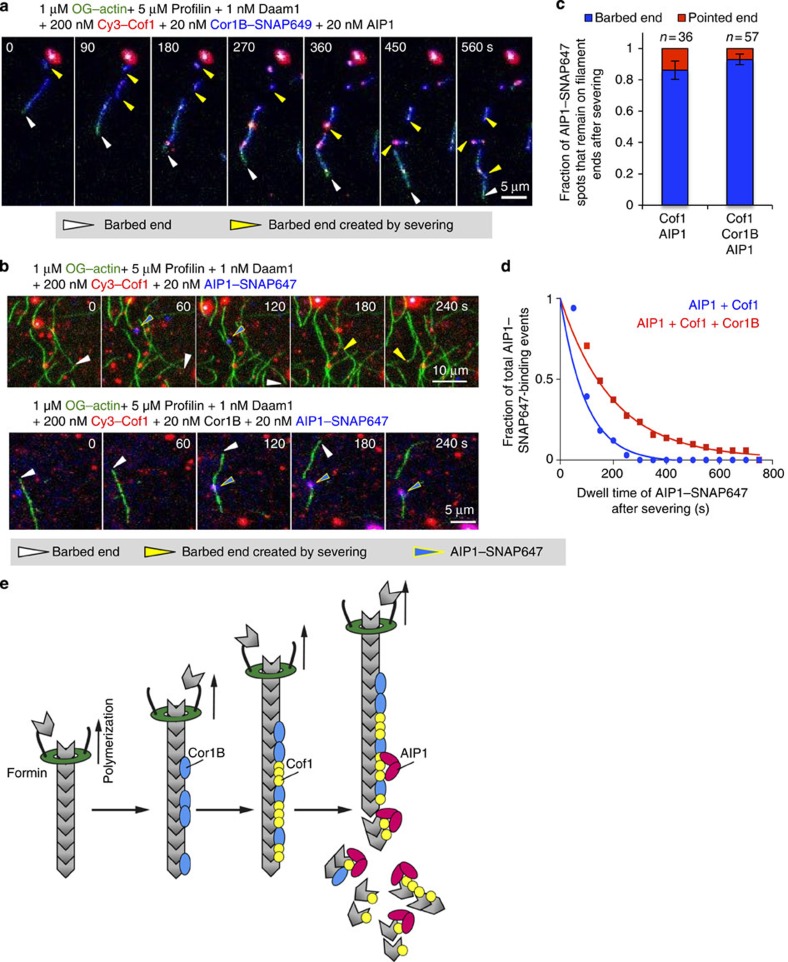
Reconstitution of rapid actin filament treadmilling. (**a**,**b**) Time points from TIRF movies showing barbed end growth stimulated by formins, concurrent with stochastic severing and capping by Cof1, AIP1 and Cor1B, leading to filament disassembly. Fluorescently labelled proteins are colour coded. White arrowheads indicate the formin-capped barbed end of the filament; yellow arrowheads indicate new barbed ends generated by severing; and yellow-outlined blue arrowheads indicate binding of AIP1–SNAP647 to filaments. (**c**) Fraction of AIP1–SNAP647 spots that remain on the barbed versus the pointed end after severing. Statistical significance and *P* value were determined by a *χ*^2^-test (not significant, *P*>0.05). (**d**) Fitted curve for the distribution of dwell times for AIP1–SNAP647 spots at newly generated barbed ends after severing in the presence and absence of Cor1B. (**e**) Model of temporally ordered disassembly by Cor1B, Cof1 and AIP1. Fast-growing filaments (bound at their barbed ends by formins) are decorated first by Cor1B. Cor1B then enhances Cof1 recruitment, leading to Cof1-stabilized filaments. Once Cof1 is present, AIP1 binds to the filaments and induces fast severing. New barbed ends generated by severing in the presence of Cor1B, Cof1 and AIP1 fail to grow, leading to net disassembly of these F-actin fragments.

Finally, we asked whether the Cor1B–Cof1–AIP1 disassembly system could be used to reconstitute filament treadmilling under assembly-promoting conditions (as found *in vivo*), in which filaments are being actively polymerized by formins at their barbed ends while at the same time undergoing stochastic and coupled severing/capping along their lengths, producing new pointed ends that disassemble. For these experiments, we used multi-wavelength TIRF microscopy in reactions containing OG–actin, profilin, formin (Daam1 construct consisting of its FH1, FH2 and C-terminal tail domains^35^), Cy3–Cof1, Cor1B–SNAP649 and AIP1. Filaments were observed to polymerize at their barbed ends at approximately fourfold accelerated rate expected for this formin in the presence of profilin^38^, while being severed along their length (Fig. 6a; Supplementary Movie 7). Cor1B–SNAP649 was observed to bind rapidly to the newly polymerized regions of the filaments, leaving only a short undecorated region behind the growing barbed end (white arrows, Fig. 6a). Cy3–Cof1 binding followed behind Cor1B–SNAP649 binding, in agreement with our kinetic analysis on preassembled filaments (Fig. 3b). Severing events correlated with sites of Cy3–Cof1 decoration (yellow arrows, Fig. 6a), and the fragments released by severing did not polymerize but rather disassembled (presumably from their pointed ends), despite the assembly-promoting conditions.

We also took advantage of this system to analyse the association of AIP1–SNAP647 with filament ends after severing because the growing barbed ends could be unambiguously identified (Fig. 6b; Supplementary Movie 8). This confirmed that AIP1–SNAP647 was recruited to sites of Cy3–Cof1 decoration on filaments, and that binding of AIP1–SNAP647 almost invariably led to severing (33/34 events for AIP1 and Cof1; 54/56 events for AIP1, Cof1 and Cor1B). Further, AIP1–SNAP647 remained bound primarily to the barbed ends of the severed filaments, both in the presence and absence of Cor1B (Fig. 5c). However, Cor1B substantially increased the average dwell time of AIP1–SNAP647 on filament barbed ends after severing, from 66.94 s for Cof1 and AIP1 (95% CI (61.41, 73.56)) to 137.4 s for Cof1, AIP1 and Cor1B (95% CI (128.3, 147.9)) (Fig. 6b,d). These results help explain why all three proteins are required for efficient obstruction of growth at barbed ends generated by severing (Fig. 5), although they do not rule out additional mechanistic contributions from Cor1B. Together, these experiments using actively growing filaments validate the key aspects of the Cor1B–Cof1–AIP1 mechanism observed above using preassembled filaments, and demonstrate that filament treadmilling can be reconstituted *in vitro* under assembly-promoting conditions.

## Discussion

In this study, we have used multi-wavelength TIRF microscopy to directly visualize the integrated actions of Coronin, Cofilin and AIP1 during the process of actin filament severing and disassembly. Our results reveal that Coronin, Cofilin and AIP1 arrive sequentially on filaments (the majority of the time in that order) and that each protein plays a key role in recruiting the next one and makes a distinct contribution to inducing highly rapid filament disassembly. These findings resolve several long-standing dilemmas about the functions and mechanisms of Coronin, Cofilin and AIP1, and provide new insights into how cells induce rapid destabilization and disassembly of actin filaments under assembly-promoting conditions. They also highlight the importance of studying the activities of groups of proteins, rather than individual ones, to define their roles and mechanisms in biological processes.

The mechanism we have defined may be used by cells to induce the highly rapid disassembly of filaments in locations such as the leading edge of cells and sites of endocytosis, where filament turnover is observed to occur in 5–20 s (refs 36, 37). Indeed, we observed complete disassembly only a few seconds after we exposed preassembled ∼10 μm long filaments to a mixture of Cor1B, Cof1 and AIP1 at their estimated cellular ratios and close to their estimated cellular concentrations. The highly rapid actin filament disassembly using near cellular concentrations of Cor1B, Cof1 and AIP1 also demonstrates that the three proteins acting together overcome many of the limitations reported for Cofilin alone (see Introduction), including delayed severing due to slow binding of Cofilin to filaments, and sub-optimal severing at high (micromolar) concentrations compared with that seen at low (nanomolar) concentrations of Cofilin^8^,^9^. By lowering the concentrations of Cor1B, Cof1 and AIP1, while maintaining their cellular ratio, we were able to quantify the contributions of each protein to the three-part mechanism, and to directly observe each protein acting on filaments during disassembly. In our system, Cof1, Cor1B and AIP1 did not cause filaments to disassemble in bursts, as described by Kueh *et al*.^31^; however, there are differences in the experimental designs which could account for the different results (see Methods).

Our experiments using labelled Coronin and Cofilin expose critical aspects of their spatiotemporal relationship, and resolve several long-standing dilemmas. First, they show that the slow binding of Cofilin to filaments is accelerated dramatically by Coronin, demonstrating that this slow step in severing can be enhanced by co-factors, and may be regulated *in vivo*. In addition, we observed that Cofilin is recruited to sites on filaments where Coronin is already bound. Precisely how this is achieved is not yet clear, but may involve Coronin altering the conformation of F-actin and/or providing additional contacts for Cofilin on filaments. Indeed, recent cryo-electron microscopy studies show that Cofilin and Coronin occupy closely juxtaposed sites on ADP-actin filaments and may be in direct contact^4^,^38^. Coronin could have additional effects on the nucleotide state of F-actin, for example, in promoting P_i_ release, but our observation that Coronin dramatically recruits Cofilin to preassembled filaments, which should be composed entirely of ADP-actin subunits, argues that Coronin most likely recruits Cofilin to ADP-actin filaments by providing additional contacts and/or inducing subtle changes in F-actin conformation that favour Cofilin binding.

A second issue resolved by our data is the seemingly paradoxical observation that, despite multiple lines of genetic evidence suggesting that Coronin promotes actin disassembly *in vivo*, Coronin alone inhibits rather than enhancing Cofilin-mediated severing *in vitro*^26^,^28^. We observed that Coronin accelerates Cofilin recruitment to filaments, both in the presence or absence of AIP1, but that without AIP1 this leads to over-decoration and hyper-stabilization of filaments by Cofilin. However, in the added presence of AIP1 it leads to strongly enhanced severing and disassembly. Thus, Coronin biochemically stimulates Cofilin-mediated actin disassembly specifically in the presence of AIP1. We also made the observation that AIP1 (without Coronin) only marginally improves the severing efficiency of Cofilin. This suggests that in addition to recruiting Cofilin to filaments, Coronin binding to filaments greatly enhances severing by AIP1 and Cof1. Moreover, after severing, Coronin increased the dwell time of AIP1 on barbed ends generated by severing, and was critical for blocking growth at those ends. Thus, Coronin makes multiple mechanistic contributions to promoting actin filament disassembly, and each of these roles is highly integrated with the functions of Cofilin and AIP1.

Our data also shed important light on the long-standing debate over the AIP1 mechanism in actin disassembly. A role for AIP1 in capping barbed ends of severed filaments has been supported by genetic interactions between AIP1 and capping protein^39^,^40^,^41^, live-imaging analysis of green fluorescent protein–AIP1 speckles at the leading edge^42^ and specific biochemical observations^21^,^25^,^39^. However, a different set of biochemical studies reported enhanced Cofilin-mediated severing in the presence of AIP1, but saw no clear evidence of barbed end capping^12^,^23^,^43^. Our observations resolve these discrepancies. We found that low concentrations of AIP1 (10–15 nM) were insufficient to cap the newly generated barbed ends. On the other hand, in the presence of Cor1B (10–15 nM), 10–15 nM AIP1 strongly enhances severing by Cofilin, but also capped the severed ends. Thus, our results show that low concentrations of AIP1 and Cor1B can together promote actin disassembly by both mechanisms, that is, enhancing severing and capping/blocking the new barbed ends generated by severing. This may explain why some previous studies (which did not include Coronin, and used different protocols and protein concentrations) did not observe barbed end capping. Using direct imaging of labelled molecules on actin filaments, we also observed that Cofilin recruited AIP1 to filament sides, and on binding invariably induced severing. AIP1 remained attached to the barbed end after severing, and the presence of Cor1B substantially increased the AIP1 dwell time on the severed end. Taken together, these data demonstrate unequivocally that AIP1 both enhances Cofilin severing and caps barbed ends generated by severing, but the efficiency of both effects of AIP1 are modulated by Coronin.

Since the strongest severing activity that we observed occurred in the combined presence of Coronin, Cofilin and AIP1, a future challenge will be to understand the structural basis for how these three proteins simultaneously interact with filaments. As mentioned above, Coronin and Cofilin appear to bind adjacent sites on filaments, so it will be important next to determine how their combined presence affects F-actin conformation, nucleotide state and mechanical properties. In addition, it is yet to be determined precisely how AIP1 binding to a Cofilin-decorated region of a filament triggers severing. This may involve AIP1 displacing one or more Cof1 molecules from a patch to generate a local discontinuity that destabilizes filaments^44^, or AIP1 displacing one of Cofilin’s two binding interactions with F-actin^45^. Finally, it will be important to study if and how Cofilin, Coronin and AIP1 work alongside other actin disassembly factors, for example, Srv2/CAP, GMF and possibly Twinfilin^16^,^18^,^46^,^47^. GMF binds with high affinity to Arp2/3 complex and stimulates filament debranching. Srv2/CAP binds to filaments, independently of Cofilin, and enhances severing not by accelerating Cofilin recruitment but instead by reducing the time from Cofilin binding to severing^16^,^17^,^18^. These mechanistic effects are distinct from, and possibly complementary to, those we have defined here for Coronin and AIP1: Coronin accelerates the recruitment of Cofilin; AIP1 is recruited to filaments by Cofilin and greatly enhances severing; and all three (Cofilin, AIP1 and Coronin) together cap severed barbed ends. Thus, each of these conserved proteins may have distinct capabilities in promoting actin filament disassembly, providing cells with a diverse tool kit with which the actin networks can be locally tuned and shaped, tailoring their architectures and dynamics for a wide range of functions.

## Methods

### Plasmids

A plasmid for expressing mouse Cor1B with a C-terminal 8His tag in mammalian cells (vector pTT5SH8Q2) was kindly provided by Dr Jim Bear (University of North Carolina). A plasmid carrying mouse AIP1 was kindly provided by Dr Naoki Watanabe (the Tohoku University), and the insert was cloned into the same mamalian expression vector as Cor1B. Plasmids for expressing Cor1B–SNAP and AIP1–SNAP were generated by cloning a SNAP-tag at the C terminus of each protein in the same expression vectors as above. The plasmid for expressing human Cof1 in *Escherichia coli* was generously provided by Dr David Kovar (the University of Chicago). For fluorescent labelling of human Cof1, a mutagenesis strategy was followed, related to that used to label yeast Cof1 (ref. 16), in which the following substitutions were introduced by site-directed mutagenesis: C139A, C147A, C39S and T63C. All constructs were verified by DNA sequencing.

### Protein purification and fluorescent labelling

Rabbit skeletal muscle actin was purified as described^48^. In brief, rabbit skeletal muscle actin was purified first by generating an acetone powder from ground muscle tissue, which was stored in aliquots at −80 °C. Aliquots of acetone powder were then pulverized using a coffee grinder, resuspended in G-buffer and cleared by low speed centrifugation. The actin was polymerized overnight and then pelleted. The pellet was disrupted by douncing, dialyzed against G-buffer for 2–3 days, and then gel filtered on a 16/60 S200 column (GE Healthcare Biosciences, Pittsburgh, PA). Column fractions were stored at 4 °C. Actin was labelled on Cys374 with either OG maleimide or DY647 maleimide (Dyomics, Jena, Germany), as described in the study by Kuhn and Pollard^49^. Briefly, monomeric actin reconstituted from an actin pellet was dialyzed against two changes of G-buffer without DTT for 1 h each. After clarification at 500*g* for 5 min, actin was polymerized by mixing an equal volume of cold 2 × label buffer (2 × =50 mM imidazole, pH 7.5, 0.2 M KCl, 4 mM MgCl2, 6 mM NaN3 AND 0.6 mM ATP). After 5 min polymerized actin was diluted to 1 mg ml^−1^ with cold 1 × label buffer, then a 10-fold molar excess of OG or DY647 maleimide was added dropwise to the actin while stirring, and the solution was stirred gently overnight. Labelled actin was clarified at 500*g* for 5 min and centrifuged at 105*g* for 2 h to pellet actin filaments. The pellet was resuspended in G-buffer by douncing, dialyzed for 2 days against two changes of G-buffer and gel filtered on a 16/60 S200 column. Peak fractions were combined and stored at −20 °C. Labelling efficiency of OG–actin was measured by absorbance at 290 and 491 nm, and extinction coefficient *E*_491_=77,800 M^−1^ cm^−1^. Labelling efficiency of DY647–actin was measured by absorbance at 290 and 653 nm, and extinction coefficient *E*_653_=250,000 M^−1^ cm^−1^. The absorption at 290 nm was corrected for background fluorescence from the dye (correction factor 0.016991 for OG–actin and 0.024 for DY647–actin). The formin Daam1 (6his-FH1-FH2-C) was inducibly expressed in yeast, and purified by sequential Ni^2+^-NTA and gel filtration chromatography steps^35^. Cor1B and AIP1 were expressed and purified from transfected HEK293T cells (ATCC). Cells were grown on plates at 37 °C under a humidified atmosphere containing 5% CO_2_ in Dulbecco’s modified Eagle’s medium, supplemented with 10% (v/v) heat-inactivated foetal bovine serum, glucose (4.5 g l^−1^), penicillin (100 U ml^−1^) and streptomycin (100 μg ml^−1^). Cells at 30–40% confluence were transiently transfected using 25 kDa linear polyethylenimine (Polysciences, Warrington, PA). About 72 h post transfection, cells were harvested in PBS, pelleted by centrifugation at 1,000*g* for 5 min and lysed by repeated freeze-thawing in 20 mM Tris/HCl pH 7.5, 150 mM NaCl, 1% (v/v) Triton X-100 and a standard cocktail of protease inhibitors (Roche, Germany). After a 30 min incubation on ice, cell lysates were cleared by centrifugation at 20,000*g* at 4 °C using an eppendorf tabletop centrifuge and incubated with Ni^2+^-NTA beads (Qiagen, Valencia, CA) for 90 min at 4 °C in the presence of 10 mM imidazole. After washing with Buffer A (20 mM Tris pH 7.5, 300 mM NaCl, 50 mM imidazole and 1 mM DTT), proteins were eluted in Buffer A supplemented with 250 mM imidazole, concentrated and purified further on a Superose 6 gel filtration column (GE Healthcare) equilibrated in Buffer B (20 mM Tris pH 8.0, 50 mM KCl and 1 mM DTT). For fluorescent labelling of SNAP-tagged proteins, the fusion proteins were bound to Ni^2+^-NTA beads, washed extensively in PBS with 1 mM DTT and incubated with a fivefold excess of benzylguanine or benzylchloropyrimidine SNAP-Surface 649 (New England Biolabs, Ipswich, MA) for 2 h at room temperature. Next, beads were washed extensively in PBS with 1 mM DTT and eluted in PBS with 250 mM imidazole. To remove free dye, proteins were exchanged into Buffer B on PD-10 columns (GE Healthcare Biosciences). Cor1B–SNAP was first labelled with SNAP-Surface Alexa Fluor 647 (New England Biolabs), but this preparation showed severe non-specific binding to slides in TIRF microscopy assays. This issue was overcome by labelling of Cor1B with SNAP-Surface 649. A labelling efficiency of 30% was obtained for Cor1B–SNAP649 and AIP1–SNAP647. Labelling efficiencies were determined spectrophotometrically using the absorbance at 650 nm and an extinction coefficient of 250,000 M^−1^ cm^−1^ for SNAP-SNAP-Surface Alexa Fluor 647 or Surface 649, combined with absorbance at 280 nm and an estimated extinction coefficient of 82,850 M^−1^ cm^−1^ for Cor1B–SNAP or 118,720 M^−1^ cm^−1^ for AIP1–SNAP. The absorption at 280 nm was corrected for background fluorescence from the dye (correction factor 0.024). Human Cof1 was expressed in BL21 (DE3) *E. coli* by growing cells at 37 °C in TB medium to log phase, then inducing expression with 1 mM isopropyl β-D-1-thiogalactopyranoside at 18 °C for 16 h. Cells were harvested by centrifugation and stored at −80 °C, then lysed by sonication in 20 mM Tris pH 8.0, 50 mM NaCl, 1 mM DTT and protease inhibitors. Lysates were cleared by centrifugation at 30,000*g* for 20 min in a Fiberlite F13-14X50CY rotor (Thermo Scientific, Rockport, Illinois), and applied to a 5 ml HiTrap HP Q column (GE Healthcare Biosciences). The flow-through containing Cof1 was collected and dialyzed into 20 mM Hepes pH 6.8, 25 mM NaCl and 1 mM DTT. Next, the protein was applied to a 5 ml HiTrap SP FF column (GE Healthcare Biosciences) and eluted with a linear gradient of NaCl (25 to 500 mM). Fractions containing Cof1 were concentrated and dialyzed into Buffer B, aliquoted, snap-frozen in liquid N_2_ and stored at −80 °C until use. Dye-labelled Cof1 was purified similarly except that the protein was eluted from the SP FF column with PBS, and then incubated with a 10-fold excess of Cy3-maleimide (GE Healthcare Biosciences) for 2 h at room temperature in the presence of 0.3 mM TCEP. Excess dye was removed by passing the protein over a PD-10 column equilibrated in Buffer B. Final labelling efficiency was 30%. Labelling efficiency was determined as described above, using absorbance at 550 nm and an extinction coefficient of 150,000 M^−1^ cm^−1^ for Cy3, combined with absorbance at 280 nm and an estimated extinction coefficient of 14,440 M^−1^ cm^−1^ for Cof1.

### TIRF microscopy

For all experiments, coverslips were first cleaned by sonication in detergent for 60 min, followed by successive sonications in 1 M KOH and 1 M HCl for 20 min each, then sonication in ethanol for at least 60 min. Coverslips were then washed extensively with H_2_O, dried in an N_2_ stream, layered with 200 μl of 80% ethanol pH 2.0, 2 mg ml^−1^ PEG–silane and 2 μg ml^−1^ biotin–PEG–silane (Laysan Bio Inc., Arab, AL) and incubated for 16 h at 70 °C. Flow cells were assembled by rinsing PEG-coated coverslips extensively with H_2_O, and then by attaching them to a flow chamber (Ibidi, Martinsried, Germany) using double-sided tape (2.5 cm × 2 mm × 120 μm) and epoxy resin. For all TIRF experiments in this study except for those shown in Fig. 6, the actin filaments were tethered. To accomplish this, flow cells were incubated for 3 min with HBSA (HEK buffer with 1% BSA), followed by 30 s incubation with 0.1 mg ml^−1^ Streptavidin in HEK buffer. Flow cells were washed HBSA and equilibrated with TIRF buffer (10 mM imidazole pH 7.4, 50 mM KCl, 1 mM MgCl_2_, 1 mM EGTA, 0.2 mM ATP, 10 mM DTT, 15 mM glucose, 20 μg ml^−1^ catalase, 100 μg ml^−1^ glucose oxidase and 0.5% methylcellulose (4,000 cP)). Reactions were initiated by rapidly diluting actin monomers (1 μM final, 10% OG labelled, 0.5% biotinylated) into TIRF buffer, followed by transferring that mixture into a flow chamber. After filaments had polymerized to lengths of ∼10–15 μm, the reaction mixture was replaced with TIRF buffer containing the indicated proteins. For assays using two differentially labelled actin preparations, filaments were first polymerized as above using 1 μM actin (10% DY647-labelled and 0.5% biotinylated), then the reaction mixture was replaced by flow-in with TIRF buffer containing 1 μM actin (10% OG labelled and 0.5% biotinylated) and the indicated proteins. To induce a manageable severing rate that allows one to follow filament growth at severed ends without over-crowding the field with filaments, different concentrations of Cof1, Cor1B and AIP1 were used in each condition. Concentrations were as follows: 250 nM Cof1 alone, 250 nM Cof1+25 nM Cor1B, 100 nM Cof1+10 nM AIP1, 75 nM Cof1+7.5 nM AIP1+7.5 nM Cor1B. For experiments in Fig. 6, non-attached filaments (no biotin-actin) were polymerized in TIRF buffer containing 2% dextran instead of 0.5% methylcellulose as the crowding agent, which minimized filament diffusion. Single- and multi-wavelength time-lapse TIRF imaging were performed using a Nikon-Ti200 inverted microscope equipped with a 150 mW Ar-Laser (Mellot Griot, Carlsbad, CA), a TIRF-objective with a numerical aperture of 1.49 (Nikon Instruments Inc., New York, NY) and an EMCCD camera (Andor Ixon, Belfast, Northern Ireland). During measurements, optimal focus was maintained by the perfect focus system (Nikon Instruments Inc.). For single-molecule photobleaching of AIP1–SNAP647, flow cells were assembled using uncoated, acid-washed coverslips and washed with HBSA. Next, 0.4 nM AIP1–SNAP647 was flowed in (in TIRF buffer without glucose oxidase and catalase), and after a 5-min incubation the adsorbed spots were exposed to continuous illumination at high laser power, and images were acquired every 0.5 s.

As noted in the Discussion, in our experiments Cof1, Cor1B and AIP1 did not cause filaments to disassemble in bursts, as reported by Kueh *et al*.^31^. However, there are two major differences in the experimental design between the studies. First, there are differences in the filament attachment mode. In most of our reactions, we incorporated a low percentage (0.5%) of biotin-actin into filaments and attached them to the surface using streptavidin. In other experiments (for example, our treadmilling assays in Fig. 6) we used untethered filaments. In both cases, we did not observe a burst of disassembly at filament ends. In constrast, Kueh and colleagues tethered filaments using filamin, raising the possibility that the bursting is promoted by this mode of attachment, and indeed Kueh *et al*. observed less bursting when filaments were tethered instead with N-ethylmaleimide-inactivated myosin. Second, the two studies used different actin-labelling strategies. Kueh and colleagues assembled filaments with a relatively high percentage of labelled actin (30%). Further, they used actin monomers that were labelled heterogenously on exposed lysines; thus, each monomer could have multiple dye molecules attached at different locations. In contrast, we used a substantially lower percentage of labelled actin (10% OG–actin), in which the dye molecules uniformly labelled on Cys^374^, so that there was only one dye molecule per actin. This strategy limits the formation of photo-induced actin dimers, which is reported to cause ‘pausing’ in actin filament depolymerization^50^.

### TIRF data analysis

All TIRF data was analysed using ImageJ software (NIH, Bethesda, MD). Before each analysis, the background was subtracted using the standard background subtraction tool (rolling ball radius 50 pixels). Severing rates were calculated by measuring the initial lengths of filaments before flow-in, and counting severing events observed during the next 300 s after flow-in of the indicated protein combinations. For binding of Cy3–Cofilin and Cor1B–SNAP, the OG–actin filaments were first traced (at 488 nm) over time using the Plot Z-axis profile tool, and then the trace was saved as a region-of-interest and used to determine the corresponding fluorescence profiles in other channels. After correction for bleed through between the Cy3 and SNAP649 channels, fluorescence in all channels was normalized to the OG–actin signal. Fluorescent traces at specific time points were obtained in a similar manner, except that they were additionally normalized to the highest signal detected in the indicated channel. The same approach was followed to monitor the change in Cor1B–SNAP649 fluorescence for three different regions of 4 × 4 pixels per filament, as shown in Fig. 3d,e, and Supplementary Fig. S4b,c. The appearance of Cor1B–SNAP649 and Cy3–Cof1 were scored by eye and confirmed by obtaining the fluorescent traces in both channels at the time of detection as described above. Subsequently, filaments were observed until the first sight of a molecule in the other channel. For kinetic analysis, measured time intervals (Fig. 4h) were fit to an exponential model using maximum likelihood methods, and fit parameter confidence intervals were estimated by bootstrapping^51^. Other statistical analyses and curve fittings (Figs 4i and 6d) were performed with Prism 5.0.

### Electron microscopy

Skeletal muscle Ca-ATP-G actin (24 μM) was polymerized by addition of inorganic salts (2 mM MgCl_2_ and 50 mM KCl) for 1 h at 25 °C. Then F-actin was diluted to 2 μM in F-buffer (50 mM KCl, 2 mM MgCl_2_, 0.2 mM EGTA, 1 mM DTT and 5 mM Tris, pH 8) and incubated for 10 min at 25 °C with one or more of the following proteins: 2 μM Cofilin, 0.2 μM AIP1 and/or 0.2 μM Cor1B. Samples were diluted another twofold in F-buffer and droplets (5–7 μl) were applied for 20–30 s to glow-discharged formvar-carbon-coated 200 mesh copper grids, then the grids were blotted to remove excess solution, negatively stained with 1% (w/v) uranyl acetate for 1 min, blotted again and allowed to air dry. Images were recorded on a CCD camera using a FEI Morgani 268 transmission electron microscope at an acceleration voltage of 80 kV and at magnifications of 5,600, 8,900 and 18,000. For measurement of filament lengths, pictures at magnifications of 5,600 or 8,900 were used, and micrographs from adjacent areas (7–15 images) on the grid were combined into one picture using Adobe Photoshop. The contour traces of each filament were measured using the ruler tool in Adobe Photoshop.

## Additional information

**How to cite this article:** Jansen, S. *et al*. Single-molecule imaging of a three-component ordered actin disassembly mechanism. *Nat. Commun.* 6:7202 doi: 10.1038/ncomms8202 (2015).

## Supplementary Material

Supplementary Information Supplementary Figures 1-5 and Supplementary References.

Supplementary Movie 1 Severing of preassembled OG-actin filaments (green) by unlabeled Cof1 (2 μM), Cor1B (0.2 μM) and AIP1 (0.2 μM).

Supplementary Movie 2 Severing of preassembled OG-actin filaments (green) by the indicated combinations of unlabeled Cof1 (150 nM), Cor1B (15 nM) and AIP1 (15 nM).

Supplementary Movie 3 Cy3-Cof1 (red) decoration of OG-actin filaments (green) in the presence of unlabeled Cor1B and/or AIP1.

Supplementary Movie 4 Binding of Cy3-Cof1 (red) and Cor1B-SNAP649 (blue) to OG-actin filaments (green).

Supplementary Movie 5 Binding of AIP1-SNAP647 (blue) only; or AIP1-SNAP647 (blue), Cy3-Cof1 (red) and Cor1B to OG-actin filaments (green).

Supplementary Movie 6 Double color actin assay to analyze polymerization at new barbed ends generated after severing. Alexa647-actin filaments (red) were first assembled, then OG-actin monomers (green), profilin and the indicated proteins were flowed in and OG-actin filament polymerization at the original barbed ends as well as at the barbed ends generated after severing was observed.

Supplementary Movie 7 Reconstitution of actin filament treadmilling of OG-actin filaments (green) in the presence of profilin, Daam1, Cor1B-SNAP649 (blue), Cy3-Cof1 (red) and unlabeled AIP1.

Supplementary Movie 8 Binding of AIP1-SNAP647 (blue) to the barbed end after severing of OG-actin filaments (green) in the presence of Cy3-Cof1 (red) and unlabeled Cor1B, profilin and an excess of OG-actin monomers. The original barbed end is indicated by a white arrow, the barbed end generated after severing by a yellow arrow.
