# Supplementary material for: Single-molecule imaging of a three-component ordered actin disassembly mechanism
Source: Nat Commun. 2015 May 21;6:7202. doi: 10.1038/ncomms8202 (PMC4443854; doi:10.1038/ncomms8202)
Supplement: Supplementary Information — Supplementary Figures 1-5 and Supplementary References. [file ncomms8202-s1.pdf]

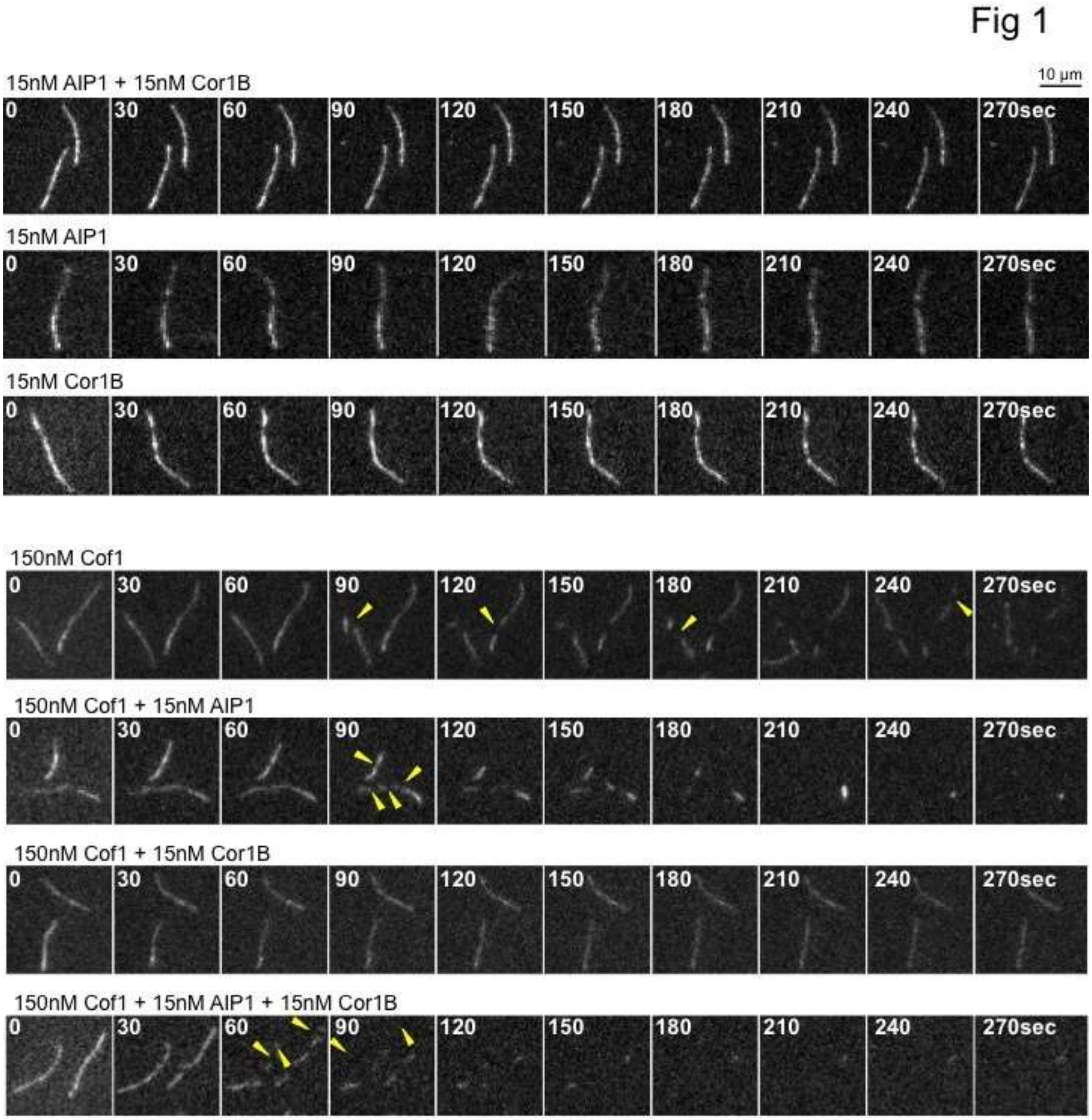

Supplementary Figure 1. TIRF microscopy analysis of actin filament severing by Cor1B, Cof1 and AIP1. Time points from TIRF microscopy movies (at a higher magnification than shown in Fig 1B) showing example OG-actin filaments used for the severing analysis shown in Fig 1D. Severing events indicated by yellow arrowheads.

Fig 2

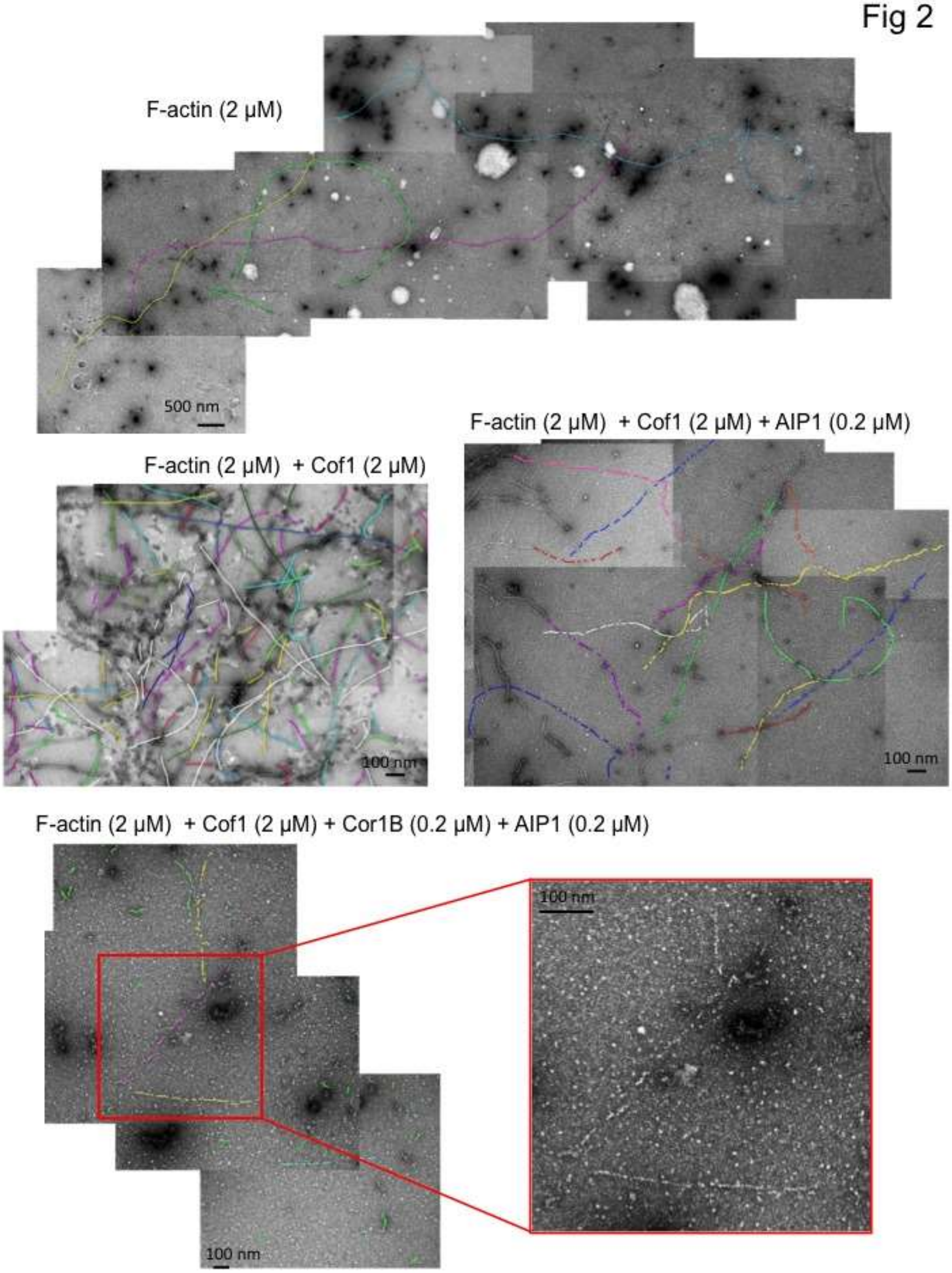

F-actin (2 µM)
500 nm
F-actin (2 µM) + Cof1 (2 µM)
100 nm
F-actin (2 µM) + Cof1 (2 µM) + AIP1 (0.2 µM)
100 nm
F-actin (2 µM) + Cof1 (2 µM) + Cor1B (0.2 µM) + AIP1 (0.2 µM)
100 nm
100 nm

Supplementary Figure 2. Example electron micrographs of severed actin filaments. Preformed filaments were incubated with the indicated proteins, negatively stained, and imaged by electron microscopy. High magnifcation images from adjacent fields were stitched together to generate a more complete view and ensure that longer filaments were accurately measured (quantification in Fig 1E). Individual filaments are marked in different colors.

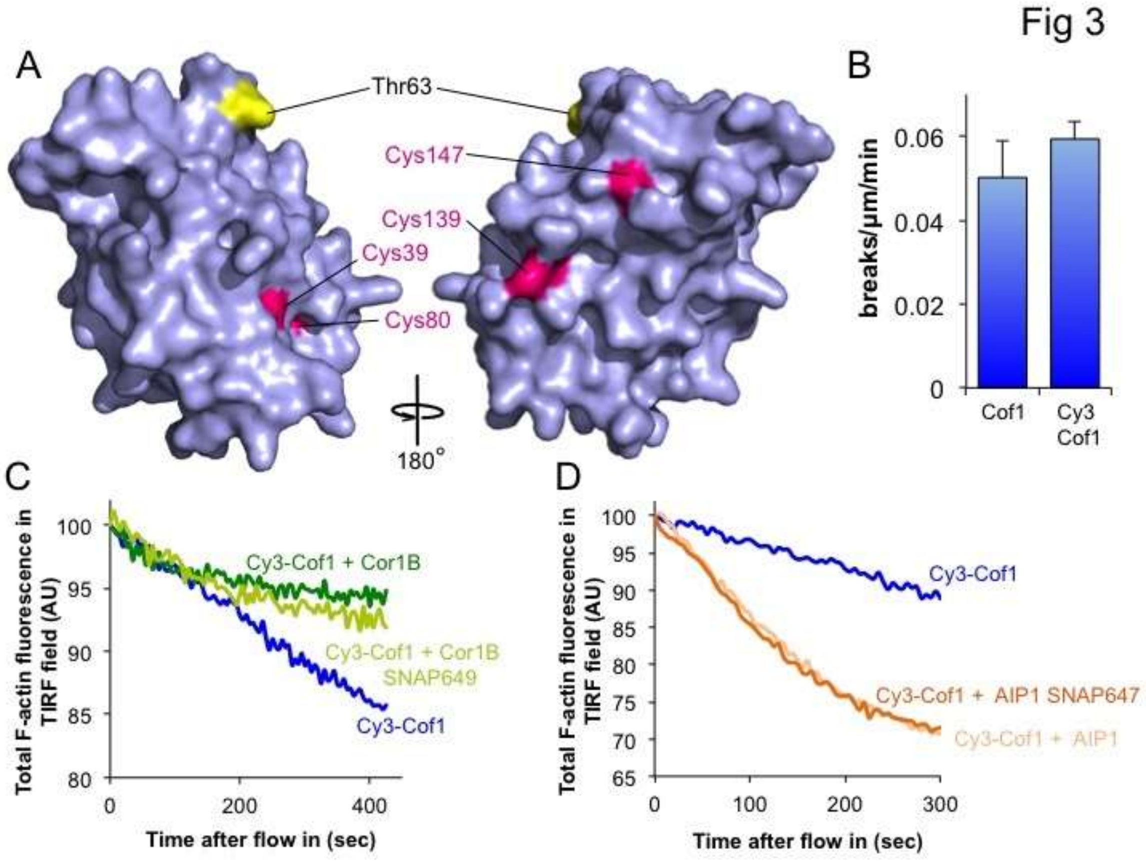

Supplementary Figure 3. Design and functional tests of Cy3-Cof1, Cor1B-SNAP649 and AIP1-SNAP647. (A) Rendered surface view of the crystal structure of human Cof1 (PDB 4BEX; [1]). Surface exposed cysteine residues mutated to alanines are shown in magenta. Thr[63] (yellow) was changed to Cys, and used for Cy3-labeling. **(B)** Comparison of actin filament severing rates induced by Cof1 and Cy3-Cof1, measured in TIRF assays 5 min after flow in. **(C)** Comparion of the effects of Cor1B-SNAP649 and unlabeled Cor1B, in the presence of Cy3-Cof1, on total OG-labeled F-actin fluorescence in TIRF assays. Filaments were first assembled, then proteins were flowed in and the change in total fluorescence (averaged for four fields of view) was measured over time. **(D)** Comparison of the effects of AIP1-SNAP647 and unlabeled AIP1, in the presence of Cy3-Cof1, on total OG-labeled F-actin fluorescence in TIRF assays, performed as in C.

Fig 4

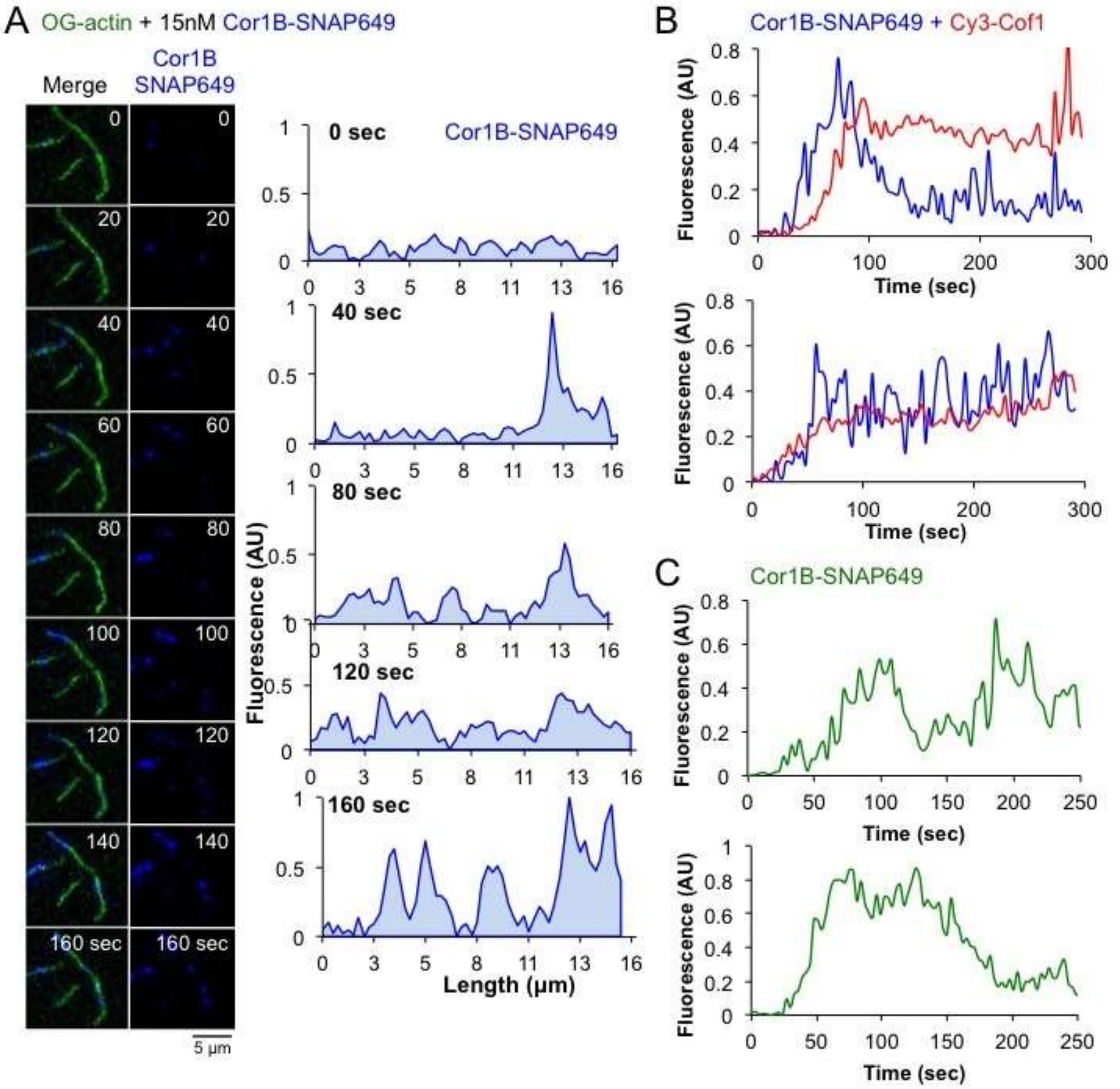

Supplementary Figure 4. Binding of Cor1B-SNAP649 in the presence and absence of Cy3-Cof1. (A) Time points from a double-color TIRF movie showing binding of Cor1B-SNAP649 to preassembled OG-labeled actin filaments after flow-in. Graphs show spatiotemporal profiles of Cor1B-SNAP649 fluorescence along the same filament at the indicated time points. **(B)** Fluorescence intensity profiles of Cor1B-SNAP649 or Cy3-Cof1 binding to a 4x4 pixel region on

a filament. (**C**) Fluorescence intensity profiles of Cor1B-SNAP649 binding to a 4x4 pixel region on a filament.

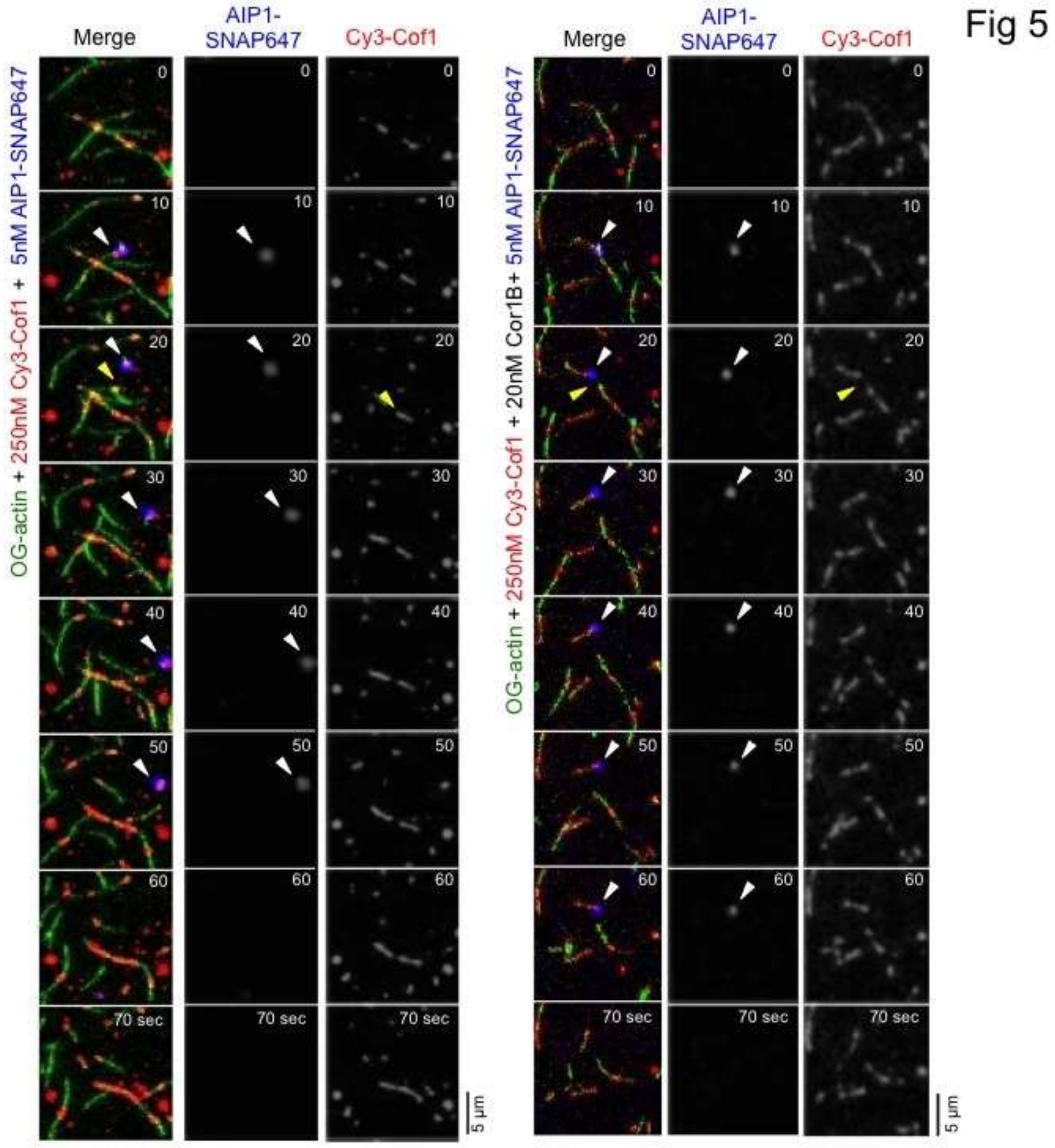

Supplementary Figure 5. Binding of AIP1-SNAP647 to actin filaments. Greyscale images of the montages shown in Fig 4A. White arrowheads indicate AIP1-SNAP647; yellow arrowheads indicate severing.

Supplementary References

1. Klejnot, M. *et al*. Analysis of the human cofilin 1 structure reveals conformational changes required for actin binding. *Acta crystallographica. Section D, Biological crystallography* **69**, 1780-1788 (2013).
